# Interferon-Gamma–Producing CD8^+^ Tissue Resident Memory T Cells Are a Targetable Hallmark of Immune Checkpoint Inhibitor–Colitis

**DOI:** 10.1053/j.gastro.2021.06.025

**Published:** 2021-10

**Authors:** Sarah C. Sasson, Stephanie M. Slevin, Vincent T.F. Cheung, Isar Nassiri, Anna Olsson-Brown, Eve Fryer, Ricardo C. Ferreira, Dominik Trzupek, Tarun Gupta, Lulia Al-Hillawi, Mari-lenna Issaias, Alistair Easton, Leticia Campo, Michael E.B. FitzPatrick, Joss Adams, Meenali Chitnis, Andrew Protheroe, Mark Tuthill, Nicholas Coupe, Alison Simmons, Miranda Payne, Mark R. Middleton, Simon P.L. Travis, Oxford Inflammatory Bowel Disease Cohort Investigators, Benjamin P. Fairfax, Paul Klenerman, Oliver Brain

**Affiliations:** 1 Translational Gastroenterology Unit, John Radcliffe Hospital, University of Oxford, Oxford, United Kingdom; 2 National Institute for Health Research Oxford Biomedical Research Centre, Oxford University Hospitals National Health Service Foundation Trust, John Radcliffe Hospital, Oxford, United Kingdom; 3 Medical Research Council Human Immunology Unit, Medical Research Council Weatherall Institute of Molecular Medicine, John Radcliffe Hospital, University of Oxford, Oxford, United Kingdom; 4 Institute of Translational Medicine, University of Liverpool, Liverpool, United Kingdom; 5 The Clatterbridge Cancer Centre National Health Service Foundation Trust, Wirral, United Kingdom; 6 Department of Cellular Pathology, Oxford University Hospitals National Health Service Foundation Trust, John Radcliffe Hospital, Oxford, United Kingdom; 7 Wellcome Centre for Human Genetics, Nuffield Department of Medicine, National Institute for Health Research Oxford Biomedical Research Centre, University of Oxford, Oxford, United Kingdom; 8 Department of Oncology, University of Oxford and Oxford Cancer Centre, Churchill Hospital, Oxford University Hospitals National Health Service Foundation Trust, Oxford, United Kingdom; 9 Translational Histopathology Laboratory, Department of Oncology, University of Oxford, United Kingdom; 10 Berkshire Cancer Centre, Royal Berkshire Hospital, Reading, United Kingdom

**Keywords:** Immunotherapy Colitis, Checkpoint Colitis, Ulcerative Colitis, Tofacitinib, cDNA, complementary DNA, DCC, anti–CTLA-4/PD-1 dual checkpoint inhibitor colitis, DCNC, anti–CTLA-4/PD-1 dual checkpoint inhibitor no colitis, FMT, fecal microbiota transplantation, HV, healthy volunteer, ICI, immune checkpoint inhibitor, IFNG, interferon gamma, irAE, immune-related adverse event, PD-1, programmed cell death protein 1, PDC, anti–PD-1-monotherapy colitis, RNASeq, RNA sequencing, scRNA, single-cell RNA, T_RM_, tissue resident memory T cell, UC, ulcerative colitis, UCEIS, Ulcerative Colitis Endoscopic Index of Severity

## Abstract

**Background & Aims:**

The pathogenesis of immune checkpoint inhibitor (ICI)–colitis remains incompletely understood. We sought to identify key cellular drivers of ICI-colitis and their similarities to idiopathic ulcerative colitis, and to determine potential novel therapeutic targets.

**Methods:**

We used a cross-sectional approach to study patients with ICI-colitis, those receiving ICI without the development of colitis, idiopathic ulcerative colitis, and healthy controls. A subset of patients with ICI-colitis were studied longitudinally. We applied a range of methods, including multiparameter and spectral flow cytometry, spectral immunofluorescence microscopy, targeted gene panels, and bulk and single-cell RNA sequencing.

**Results:**

We demonstrate CD8^+^ tissue resident memory T (T_RM_) cells are the dominant activated T cell subset in ICI-colitis. The pattern of gastrointestinal immunopathology is distinct from ulcerative colitis at both the immune and epithelial-signaling levels. CD8^+^ T_RM_ cell activation correlates with clinical and endoscopic ICI-colitis severity. Single-cell RNA sequencing analysis confirms activated CD8^+^ T_RM_ cells express high levels of transcripts for checkpoint inhibitors and interferon-gamma in ICI-colitis. We demonstrate similar findings in both anti–CTLA-4/PD-1 combination therapy and in anti–PD-1 inhibitor-associated colitis. On the basis of our data, we successfully targeted this pathway in a patient with refractory ICI-colitis, using the JAK inhibitor tofacitinib.

**Conclusions:**

Interferon gamma–producing CD8^+^ T_RM_ cells are a pathological hallmark of ICI-colitis and a novel target for therapy.

## Graphical abstract

**Figure:**
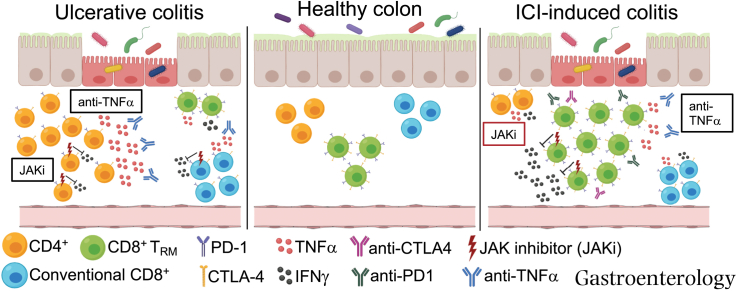

## Teaser

We present an analysis of the immunopathology in checkpoint-inhibitor colitis, a common adverse effect of cancer immunotherapy. We used our findings to successfully identify a novel therapy for a case of refractory colitis.

See Covering the Cover synopsis on page 1079; See editorial on page 1106.

**What You Need to Know**

**Background and Context**

ICI-colitis is a common adverse effect of checkpoint inhibitors, can mimic IBD, and currently has empirically derived treatment guidelines.

**New Findings**

We identify both unique and overlapping immunopathology in ICI-colitis and UC. CD8^+^ T_RM_ cells are the key effector cells in ICI-colitis. T_RM_ cells strongly express checkpoint proteins and IFNG. We present the first PD1-inhibitor–associated colitis single-cell analysis that demonstrates consistent T_RM_ cell activation. IFNG-JAK-STAT activation identified tofacitinib as a potential therapy, although IFNG blockade could negatively affect oncological response.

**Limitations**

This is a small human cohort study. Further investigation will be required to understand the role of the microbiome in T_RM_ cell activation and the safety of JAK inhibition.

**Impact**

This analysis of CD8^+^ T_RM_ cells and associated immune pathways in ICI-colitis provides a basis for targeted therapy development. We provide an immunologic rationale for the use of JAK inhibitor therapy in refractory ICI-colitis.

Immune checkpoint inhibitors (ICIs) are revolutionizing the treatment of melanoma and other cancers but come at the cost of immune-related adverse events (irAEs). These irAEs commonly affect the gastrointestinal tract, with those receiving combination anti–CTLA-4 and PD-1 therapy displaying increased rates of ICI-colitis (32%–37%) compared to those treated with anti–PD-1 monotherapy (4%–6%).^1^^,^^2^ There is a higher incidence of ICI-diarrhea (44%),^1^ probably due to unconfirmed colitis. ICI-colitis results in the greatest overall mortality of irAE, although other rarer toxicities (eg, myocarditis) have lower individual survival rates.^3^

Current management for ICI-colitis includes systemic corticosteroids and subsequent anti-TNFα therapy (infliximab) for inadequately controlled disease.^4^ Alternative therapies include anti-α4β7 integrin (vedolizumab)^5^ and fecal microbiota transplantation (FMT).^6^ These therapeutic approaches are empirically derived from the treatment of idiopathic inflammatory bowel disease (IBD), without an understanding of how analogous this newer entity is to more classical forms of colitis. Refractory cases of ICI-colitis occur, resulting in steroid toxicity and, on occasion, colectomy. It is anticipated that greater insight into the mechanisms underlying ICI-colitis will lead to more targeted treatments. ICI-colitis is heterogeneous, but can mimic UC and, less commonly, Crohn’s disease.^7^ We opted to use UC as the external disease control, as both conditions typically affect the rectum and/or sigmoid colon and are amenable to flexible sigmoidoscopy.

**Figure 1:**
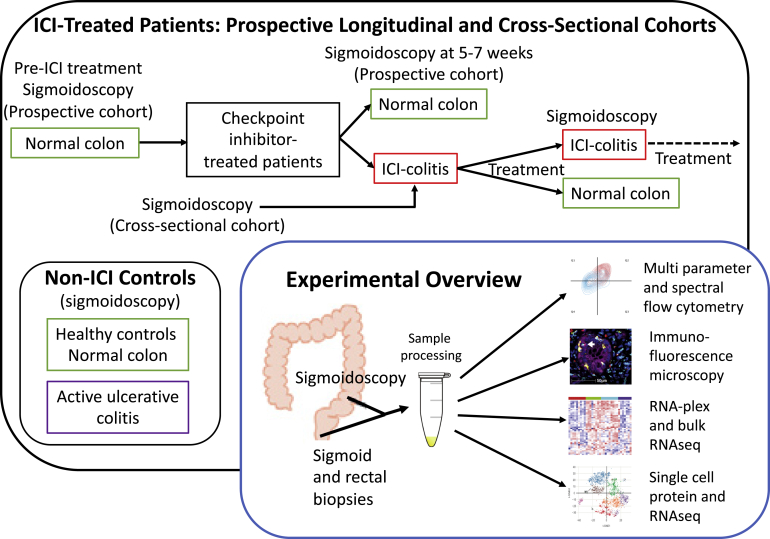
Study design.

At the outset of this study, little was known about the cellular and molecular pathogenesis of ICI-colitis. The available data suggested that anti–CTLA-4–associated colitis is associated with CD8^+^ T cells^8^ and an up-regulation of Th1 and Th17 effector pathways, including interferon-gamma (*IFNG*).^9^ We previously demonstrated that anti–CTLA-4/PD-1 colitis is associated with high levels of activated (HLA-DR^+^CD38^+^) memory CD8^+^ T cells,^10^ and lower proportions of regulatory T cells compared with UC.^10^ We hypothesized that CD8^+^ T_RM_ cells are implicated in the pathogenesis of ICI-colitis, postulating that they would become activated in an off-target consequence, and sought to understand the signaling pathways involved (Figure 1).

T_RM_ cells are specialist lymphocytes enriched at mucosal sites, including the gut,^11^ and display minimal circulation. CD8^+^ T_RM_ cells classically express CD69 and CD103, and play an important role in mucosal immunity (reviewed in Sasson et al^12^). They are implicated in the pathogenesis of autoimmune skin conditions.^13^ T_RM_-like tumor infiltrating lymphocytes also mediate anti-tumor responses,^14^^,^^15^ and a higher proportion of T_RM_-like tumor infiltrating lymphocytes correlates with disease-free survival.^16^^,^^17^ T_RM_-like tumor infiltrating lymphocytes express high levels of checkpoint proteins,^18^, ^19^, ^20^ which appear to act primarily as negative regulators rather than markers of exhaustion.

Recent data utilized single-cell technology to identify cytotoxic CD8^+^ T cells as the main pathogenic gastrointestinal population in anti–CTLA-4/PD-1 colitis.^21^ It was inferred through T cell receptor sequence analysis that these may derive, in part, from T_RM_ cells. Up-regulation of both *IFNG* and *TNFA* signaling pathways was identified in the CD4^+^ and CD8^+^ T cells of patients with ICI-colitis, acting on the myeloid cellular compartment.^21^ Our study supports and extends the prior analysis via a broader range of experimental techniques. We also provide a direct comparison with idiopathic UC and include analysis of anti–PD-1 monotherapy colitis and anti–CTLA-4/PD-1 gastritis. Our data taken together directly implicate *IFNG-*expressing CD8^+^ T_RM_ cells as a major pathogenic T cell population in the colon.

Finally, on the basis of our laboratory data, we predicted that tofacitinib would be an effective therapy in a case of refractory anti–PD-1 colitis and used this successfully. These data, consistent with the subsequently published case reports of successful tofacitinib therapy,^22^^,^^23^ provide compelling insights into the underlying pathogenic mechanisms in this clinical setting and novel pathways to target therapeutically.

## Materials and Methods

### Subjects

We studied patients with anti–CTLA-4/PD-1 colitis (dual checkpoint inhibitor colitis [DCC]; n = 15), anti–CTLA-4/PD-1 treated with no colitis (DCNC; n = 10), anti–PD-1 colitis (PDC; n = 6), anti–PD-1–treated with no colitis (n = 5), active UC (disease flare assessment; n = 10), and healthy volunteers (HVs; n = 22). The patients with UC and ICI-colitis are reasonably matched in terms of inflammation severity and previous and current therapy but do have a longer median acute flare duration in the UC cohort (see Supplementary Table 1), and the usual caveats must apply when interpreting real-world human data from subjects that are not perfectly aligned. All patients provided written informed consent for participation (Oxford GI Illness Biobank 16/YH/0247 or PRISE [Predicting Immunotherapy Side Effects] study, London-Surrey Research Ethics Committee: REC18/LO/0412). This consent enabled invitation for sigmoidoscopy pre-ICI, at week 5–7 post ICI treatment and at the development of colitis symptoms (see Figure 1). Colitis disease activity is comparable across UC and ICI-colitis. The duration of active colitis and the treatment at the time of sampling are detailed in Supplementary Table 1. Rectal and sigmoid biopsies were protocolized. Individual patient samples studied in each experiment are shown in Supplementary Table 2.

The clinical characteristics of patients with anti–CTLA-4/PD-1–associated gastritis (n = 4) and HV controls (n = 7) are shown in Supplementary Table 3.

### Isolation of Mononuclear Cells From Gastrointestinal Tissue

Colonic biopsies were placed in RPMI media containing penicillin and streptomycin and 10% fetal calf serum. Biopsies underwent enzymatic and mechanical digestion with 1 mg/mL Collagenase D (Sigma-Aldrich) and 100 μg/mL DNAse I (ThermoFisher) and were shaken at 37°C for 1 hour. Biopsies were dissociated using gentleMACS Dissociator (Miltenyi Biotech) and passed through a 70-μm strainer.

### Flow Cytometry

Flow cytometry was performed on freshly isolated mononuclear cells using a near-infrared live/dead stain (Invitrogen) and an initial monoclonal antibody panel (see Supplementary Methods) was performed on a 3-laser LSR Fortessa X-20 (BD Biosciences). An extended T_RM_ cell phenotyping monoclonal antibody panel (see Supplementary Methods) was performed on an Aurora spectral analyzer (Cytek).

Data were analyzed using FACSDIVA software, version 8.0.1 (BD Biosciences). Gating strategies are shown in Supplementary Figure 2*A*. Lymphocyte populations are reported as a proportion of parent populations.

### Statistics

Differences between groups were determined using the unpaired nonparametric Mann-Whitney test. Correlation analysis was performed using the nonparametric Spearman test. All analyses were performed using SPSS software (IBM, Armonk, NY). Medians and interquartile ranges are reported throughout. A *P* value < .05 was considered statistically significant. When multiple comparisons were performed, a Bonferroni correction was made (see figure legends).

### Multiplex Immunofluorescence Microscopy

Multiplex immunofluorescence staining was carried out on 4-μm formalin-fixed paraffin embedded sections using the OPAL protocol (AKOYA Biosciences) on the Leica BOND RX^m^ autostainer (Leica Microsystems). Six consecutive staining cycles were performed using primary antibody-Opal fluorophore pairings. Whole slide scans and multispectral images were obtained on the AKOYA Biosciences Vectra Polaris. Batched analyzed multispectral images were fused in HALO (Indica Labs) to produce a spectrally unmixed reconstructed whole tissue image.

### Nanostring RNA Plex

Targeted gene expression was measured using 150 μg of RNA extracted from pinch biopsies and a 770-gene human autoimmune profiling panel with a custom 10-gene spike in set (Supplementary Methods). Samples were analyzed on an nCounter Sprint profiler with downstream analysis using nSolver freeware (Nanostring), Gene Set Enrichment Analysis (Broad Institute), and R studio (Boston).

### Bulk RNA Sequencing

Bulk RNA sequencing (RNASeq) analysis was performed using 900 ng per sample of RNA extracted from pinch biopsies and the GRCH37.EBVB95-8wt reference genome. Total RNA was converted to complementary DNA (cDNA) with second-strand cDNA incorporating a 2'-deoxyuridine 5'-triphosphate. cDNA was end-repaired with PolyA tails and was adapter-ligated. Sequencing was performed on a NovaSeq6000 (Illumina). Bulk RNASeq was analyzed using Partek Flow software. Library generation and sequencing were performed at the Oxford Genomics Centre. Data analysis was performed according to published standards.^24^, ^25^, ^26^, ^27^

### 10X Genomics Library Preparation and Sequencing

Single-cell (sc)RNASeq libraries were generated using 10X Genomics Chromium scRNA Reagents Kits (v1 Chemistry). Live CD45^+^ cells were sorted using a FACSAriaIII cell sorter (BD Biosciences) and resuspended in phosphate-buffered saline with 0.04% bovine serum albumin at approximately 1000 cells/μL and loaded onto 2 lanes of the Chromium Controller. Captured cell number was 5876. Library quality and concentration was determined using a TapeStation (Agilent) and Qubit 2.0 Fluorometer (Thermo Fisher). Libraries were sequenced on an Illumina HiSeq 4000 to a mean depth of 64,000 mean reads/cell. Library generation and sequencing were performed at the Oxford Genomics Centre.

### Droplet-Based (10X Genomics) Single-Cell RNA Sequencing Data Analysis

FastQ generation, read alignment, barcode counting, and UMI counting was performed using the Cell Ranger Pipeline, version 2.2.0. Downstream processing steps were performed using Seurat, version 2.3.4. Genes expressed in fewer than 10 cells were removed. Cells with a local minimum of the UMI distribution to the left of the mode UMI count, <500 genes, and >10,000 UMIs, > 2500 genes, and/or > 10% mitochondrial reads were removed. Data were log-normalized and scaled, with cell–cell variation due to UMI counts, percent mitochondrial reads, and S and G2M cell cycle scores regressed out.

### Genetic De-Multiplexing Single-Cell RNA Sequencing

Demultiplexing scRNASeq was run with the inferred genotypes from the bulk RNASeq data that we have sequenced as part of this same project. We used the GATK variant calling pipeline on the samples included in each pool (GX06/GX18) and fed that to demuxlet as described in Kang et al.^28^

### Single-Cell RNA Sequencing Data Processing and Quality Control

Cellranger (version 3.0.2) mkfastq was applied to the Illumina BCL output to produce FASTQ files. Cellranger count was then applied to each FASTQ file to produce a feature barcoding and gene expression library. Cellranger aggr was used to combine samples for merged analysis.

We applied *scater* package to filter out single-cell profiles that were outliers for any metric, as low-quality libraries.^29^ Data analysis was performed according to published standards.^30^, ^31^, ^32^, ^33^ All datasets used and additional scripts are available online (https://bitbucket.org/Fairfaxlab/prise-sarah-sassion/src/master/).

### Identification T Cell Clusters

We used the area under the curve to calculate whether a T cell reference gene set was enriched within the expressed genes for each cell.^34^ We used the Human Protein Atlas database reference gene list for T cells, downloaded from https://www.proteinatlas.org/humanproteome/blood/blood+cells+summary, cell type group enriched genes. the repository is provided as a supplementary file (repository_reference_gene_sets.txt).

### Single-Cell Protein and RNA Sequencing Expression

Live CD45^+^CD3^+^ T cells were sorted using a FACSAriaIII cell sorter (BD Biosciences). A total of 46,000 T cells were sorted and stained with a cocktail of 70 oligo-conjugated AbSeq antibodies (BD Biosciences; see Supplementary Table 4) for 45 minutes at 4ºC. Cells were then washed to remove residual unbound AbSeq antibodies and loaded onto 3 BD Rhapsody cartridges (BD Biosciences) for single-cell capture.

### Complementary DNA Library Preparation and Sequencing

Single-cell capture and cDNA library preparation were performed using the BD Rhapsody Express single-cell analysis system (BD Biosciences) and a customized T cell expression panel (Supplementary Table 5), according to the manufacturer’s instructions (for further details including data analysis and quality control, see Supplementary Methods).

## Results

### The Majority of Activated Colon CD8^+^ T Cells in Anti–CTLA-4/PD-1 Colitis Are Tissue Resident Memory T Cells

**Figure 2:**
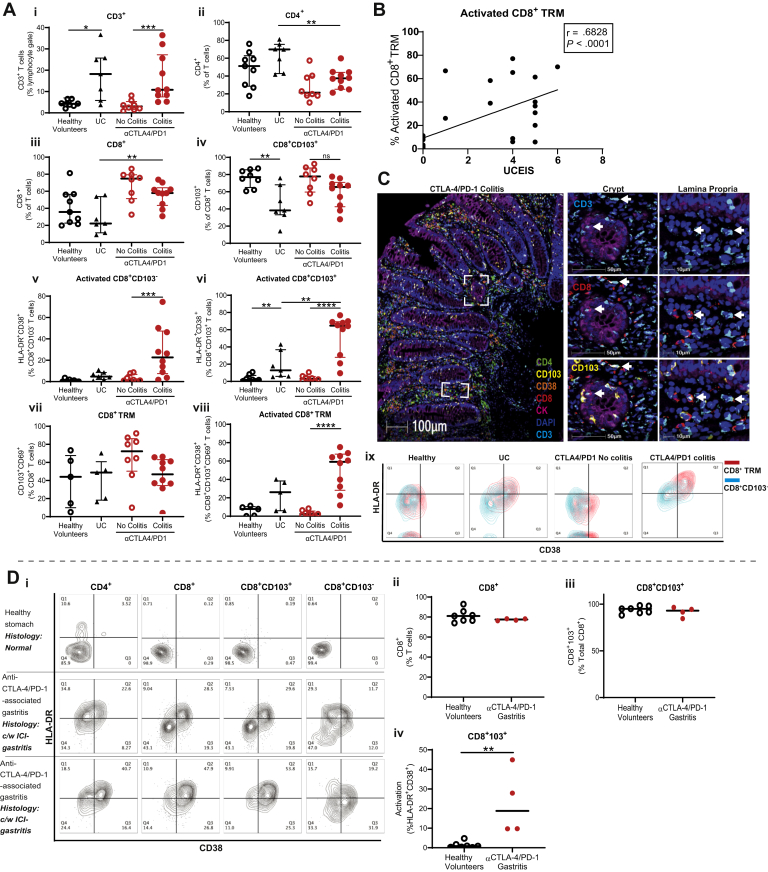
CD8^+^ T_RM_ cells predominate in ICI-colitis and their activation correlates with endoscopic and histologic findings. Mononuclear cells from colonic biopsies from HVs (n = 8), active UC (n = 7), DCC (n = 12), and DCNC (n = 8). Flow cytometry demonstrates (*Ai–iii*) DCC is associated with a CD3^+^ T cell lymphocytosis, and CD8^+^ T cells predominance. (*iv*–*vi*) The majority of CD8^+^ T cells express tissue-residency marker CD103, with higher activation in colitis than CD8^+^103^*–*^ counterparts. (*vii–viii*) The proportion of CD8^+^CD69^+^CD103^+^ T_RM_ cells does not significantly differ across disease states; however, activation is highest in the DCC group. ∗*P* < .02; ∗∗*P* < .01; ∗∗∗*P* < .001; and ∗∗∗∗*P* < .0001 by Mann-Whitney test with Bonferroni correction. (*ix*) Co-expression of activation markers CD38 and HLA-DR are highest in patients with UC and DCC with CD8^+^CD69^+^CD103^+^ T_RM_ cells (*red*) displaying higher expression of these markers than CD8^+^CD103^*–*^ nonresident T cells (*blue*). Live CD45^+^CD8^+^ T cells are shown. (*B*) Proportion of activated CD8^+^ T_RM_ cells positively correlates with anti*–*CTLA-4/PD-1 colitis severity and measured by UCEIS (Spearman correlation). (*C*) Multiplexed spectral microscopy of a patient with DCC. Colocalization of CD3, CD8, and CD103 is demonstrated in both gastrointestinal crypts and in the lamina propria. CK, cytokeratin; DAPI, 4′,6-diamidino-2-phenylindole nuclear stain. Data representative of 3 experiments. (*D*) Live, singlet CD45^+^CD3^+^ T cells are displayed. (*I*) Cellular activation of T cells (*top right quadrant*) in healthy stomach and patients with anti*–*CTLA-4/PD-1 gastritis. In both health and anti*–*CTLA-4/PD-1 gastritis the majority of T cells are (*ii*) CD8^**+**^ with **(***iii*) T_RM_ cell phenotype. (*iv*) Increased cellular activation is present in anti*–*CTLA-4/PD-1 gastritis compared with healthy stomach.

We previously demonstrated that the majority of T cells in the colon of ICI-colitis were CD8^+^.^10^ We sought to determine whether these CD8^+^ T cells had a T_RM_ cell phenotype. We found that both UC and DCC groups are associated with increased proportions of CD3^+^ T cells in the affected tissue compared with healthy gut and DCNC groups, respectively (Figure 2*Ai*). Compared with UC, DCC is associated with proportionately fewer CD4^+^ T cells and more CD8^+^ T cells (Figure 2*Aii–iii*). The proportion of CD8^+^CD103^+^ T cells is lower in active UC compared with HVs (Figure 2*Aiv*). Patients with DCC have a very high proportion of activated CD8^+^CD103^+^ T cells, as defined by the co-expression of HLA-DR and CD38 (median 65% of CD8^+^CD103^+^ T cells), and this is higher than in DCNC (3%; *P* < .0001) and UC (13%; *P* < .01) groups (Figure 2*Avi*). There is some activation of CD8^+^103^–^ “non–tissue-resident” T cells in DCC compared with DCNC; however, the proportion is much lower than in the CD103^+^ subset (Figure 2*Av*). We used a more stringent definition of CD8^+^ T_RM_ cells, that is, co-expression of CD69 and CD103 to confirm high levels of cellular activation of CD8^+^ T_RM_ cells in DCC compared with DCNC (Figure 2*Avii–viii*).

Higher cellular activation of CD103^+^CD69^+^ CD8^+^ T_RM_ cells compared to CD103^*–*^ CD8^+^ T cells was detected across all patient groups and is represented in Figure 1*Aix*.

### The Proportion of Activated Colon CD8^+^ Tissue Resident Memory T Cells Correlates Longitudinally With Clinical and Endoscopic Findings

**Supplementary Figure 1:**
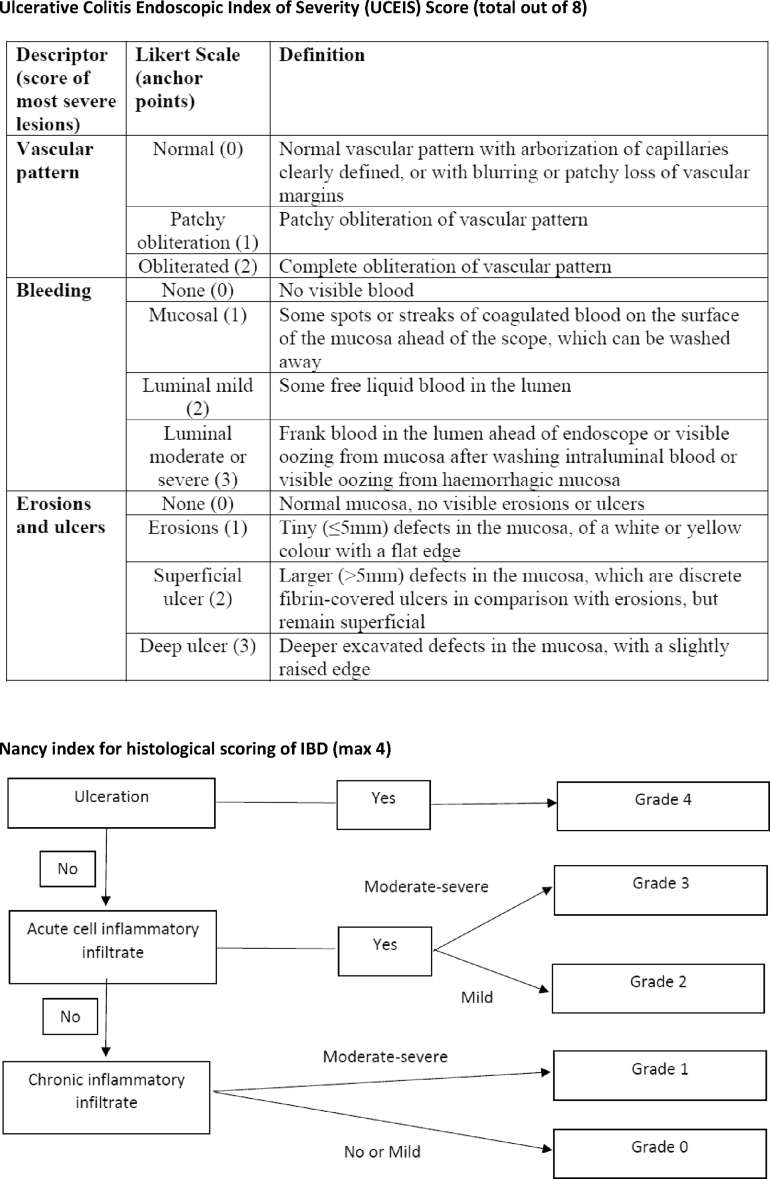
UCEIS and Nancy Index system for the scoring of UC.

We investigated whether the proportion of activated CD8^+^ T_RM_ cells in the colon was an accurate biomarker of the presence of DCC and response to therapy. Overall, CD8^+^ T_RM_ cell activation positively correlates with endoscopic severity of DCC using the Ulcerative Colitis Endoscopic Index of Severity (UCEIS) score (Figure 2*B*). UCEIS score details are provided in Supplementary Figure 1. We previously reported that UCEIS can be used as an objective endoscopic marker of ICI-colitis clinical outcomes.^2^ We confirmed by multiplexed spectral fluorescence microscopy that in DCC, CD8^+^ T_RM_ cells reside both in gastrointestinal crypts and in the lamina propria (Figure 2*C*).

We investigated whether activation of CD8^+^ T_RM_ cells was a phenomenon specific to ICI-colitis or was evident in other forms of irAEs, such as ICI-gastritis (Figure 2*Di*). CD8^+^ T_RM_ comprise the majority of T cells in the gastric mucosa in both health and anti–CTLA-4/PD-1 gastritis (Figure 2*Dii–iii*). The proportion of activated CD8^+^ T_RM_ cells was low in health (<1%; Figure 2*Div*) and increased in anti–CTLA-4/PD-1 gastritis (30%–51%; Figure 2*Div*).

**Supplementary Figure 2:**
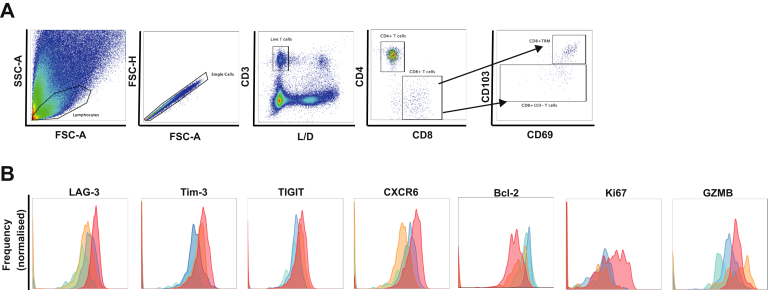
(*A*) Flow cytometric gating strategy. (*B*) CD8^+^CD69^+^CD103^+^ T_RM_ cells in anti-CTLA-4/PD-1 colitis (*red*) displayed high levels of checkpoint molecules LAG-3, Tim-3, TIGIT, and chemokine receptor CXCR6. Intracellularly they had high Ki-67 and granzyme-B and low levels of anti-apoptotic protein Bcl-2. Anti-CTLA-4/PD-1–associated colitis non-T_RM_ cells (*orange*), CTLA-4/PD-1 treatment with no colitis CD8^+^ T_RM_ cells (*dark blue*) and non-T_RM_ cells (*light blue*). *Histograms* based on live CD45^+^CD8^+^ T cells. Data are representative of 5 experiments.

We performed an extended flow cytometric panel to further characterize the CD8^+^ T_RM_ cells in anti–CTLA-4/PD-1 colitis (Supplementary Figure 2*B*).

### Anti–CTLA-4/PD-1 Colitis Has a Transcriptome Distinct From Ulcerative Colitis With Up-Regulated Interferon-Gamma Signaling

**Figure 3:**
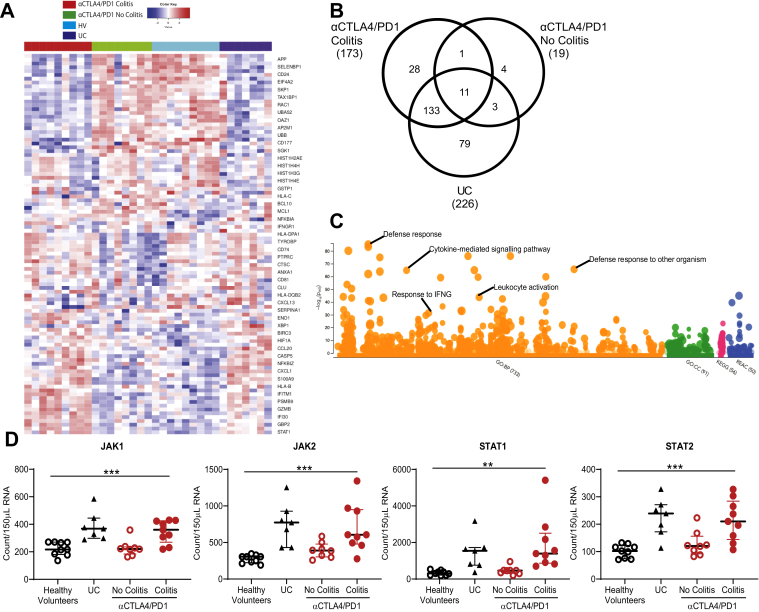
Targeted gene panel analysis of ICI-colitis includes unique and common features compared with UC and high expression of the IFNG signaling pathway. A 780-gene Nanostring analysis of colonic biopsy RNA from HVs (n = 8), patients with active UC (n = 5), DCC (n = 9), and DCNC (n = 8). (*A*) Heatmap of top 50 differentially expressed genes. (*B*) Number of genes up-regulated ≥2-fold compared with HVs demonstrates 173 of up-regulated genes in DCC, only 12 of 173 are also up-regulated in the DCNC group (limited on-treatment effect); 144 of 173 genes are common between DCC and UC, 28 of 173 genes are unique to DCC. (*C*) *Manhattan plot* indicating significantly up-regulated pathways, including response to IFNG. The complete list is provided in Supplementary Table 4. (*D***)** RNA expression of canonical markers of IFNG signaling JAK1, JAK2, STAT1, and STAT2 are higher in DCC and UC groups compared with healthy controls and DCNC groups. ∗∗*P* < .01 and ∗∗∗*P* < .001 by 1-way analysis of variance.

We performed a 780-gene autoimmune profiling panel using colonic RNA to determine unique and common features among DCC, DCNC, and UC groups. A heatmap of the top 50 defining features demonstrates that DCC is associated with up-regulated *STAT1, GBP2, IFI30, GZMB, PSMB9, IFITM1, HLAB, S100A9*, and *CXCL1* (Figure 3*A*).

We identified 259 genes that are up-regulated more than 2-fold across the DCC, DCNC, and UC groups compared to HVs (Figure 3*B*). Of the 173 genes up-regulated in DCC, only 12 of 173 are common to the DCNC group, indicating these changes are not simply an on-treatment effect. Of the 173 genes up-regulated in DCC, 144 of 173 are in common with UC, and 29 are unique (Figure 3*B*).

Exploration of the 173 up-regulated genes in DCC using g:Profiler pathway analysis highlights a number of biological pathways (Figure 3*C*). The top 30 pathways include defense response and response to external biotic stimulus and cytokine-mediated pathways. The most pathway-specific results include a response/cellular response to IFNG (Figure 3*C*; Supplementary Table 6). We confirm that the canonical JAK/STAT components of IFNG signaling are up-regulated in anti–CTLA-4/PD-1 colitis compared to controls (Figure 3*D*).

**Supplementary Figure 3:**
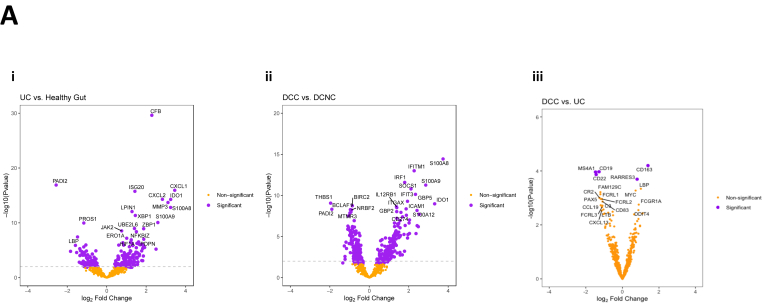
Data generated from a 780-gene Nanostring experiment analyzing RNA extracted from the gastrointestinal tract of HVs (n = 8), patients with active UC (n = 5), anti–CTLA-4/PD-1 colitis (DCC; n = 9) and anti–CTLA-4/PD-1 treated with no colitis (DCNC; n = 8) are shown. *Plots* show differentially expressed genes in (*i*) UC vs HV and (*ii*) DCC vs DCNC and DCC vs UC.

Volcano plots showing differentially expressed genes in UC vs HV, DCC vs DCNC, and DCC vs UC are shown in Supplementary Figure 3. Up-regulated genes common to DCC and UC include *S100A8*, *S100A9*, and *IDO1*. DCC is associated with lower expression of canonical B cell markers CD19, MS4A1(CD20), and CD22 compared to UC.

### Bulk RNA Sequencing Analysis Confirms a Distinct Transcriptome for Anti–CTLA-4/PD-1–Associated Colitis Enriched for Interferon-Gamma Signaling

**Figure 4:**
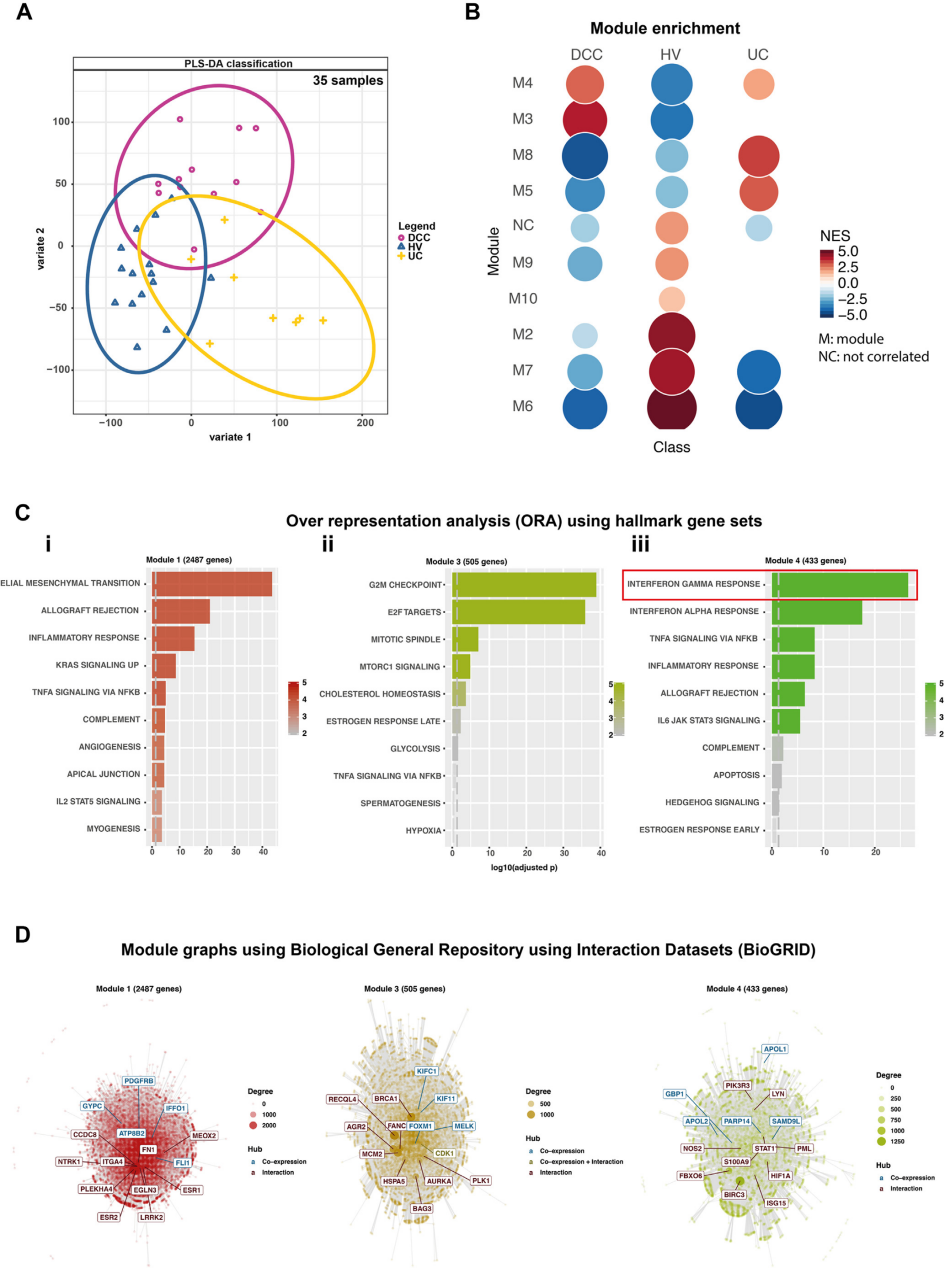
Bulk RNASeq analysis confirms anti*–*CTLA-4/PD-1 colitis has a transcriptome distinct from UC with IFNG signaling stronger than TNFα signaling. Bulk RNASeq data generated from total RNA extracted from patients with DCC, those with active UC and HVs are shown. (*A*) Partial least squares-discriminate analysis (PLS-DA) demonstrate the divergent transcriptome of DCC and UC. (*B*) Module enrichment analysis demonstrated overexpression of hallmark gene “modules” 3 and 4 in DCC. (*C*) Over-representation analysis demonstrates the over-represented genesets in modules 1, 3, and 4. Over-expressed pathways include *IFNG* signaling (*box*),which was more highly expressed than TNFα signaling. (*D*) Co-expression and interaction of genes in modules 1, 3, and 4 as determined by Biological General Repository using Interaction Datasets (BioGRID). *Blue* indicates co-expressed genes; *brown* indicates gene interaction; and *green* indicates gene co-expression and interaction.

RNASeq analysis from bulk RNA extracted from colonic biopsies confirms the transcriptomic signature associated with DCC is distinct from UC (Figure 4*A* and *B*). As opposed to the Nanostring panel, which selects for genes expressed by lymphocytes, the bulk RNASeq analysis is predominated by epithelial signals. Analysis of modular hallmark gene sets demonstrates patients with DCC have highly expressed *IFNG* response, in excess of *TNFA* signaling (Figure 4*C*).

### Single-Cell RNA Sequencing Confirms Anti–CTLA-4/PD-1 Colitis Is Associated With High Proportions of Activated CD8^+^ Tissue Resident Memory T Cells That Express Transcripts for CTLA-4, PD-1, TIGIT, TIM-3, LAG-3, and Interferon-Gamma

**Figure 5:**
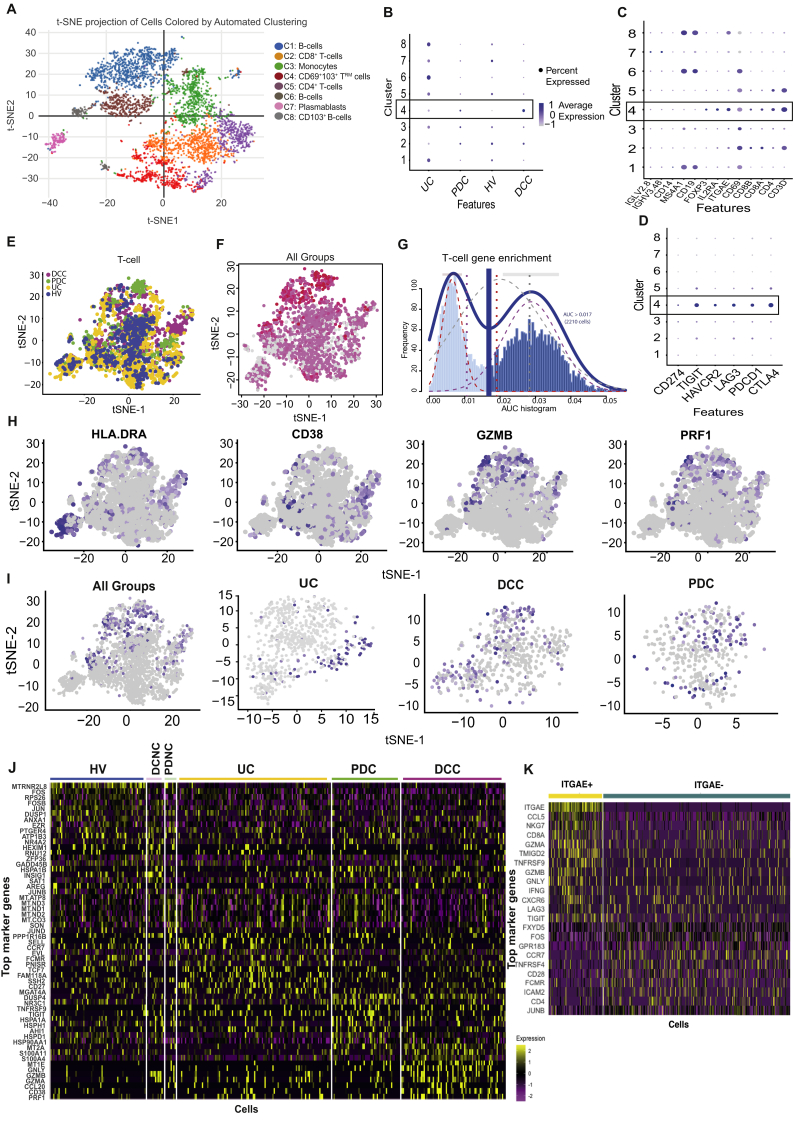
CD8^+^ T_RM_ cells in ICI-colitis express high proportions of checkpoint inhibitors, cellular activation/cytotoxicity markers and *IFNG*. scRNASeq analysis of 5876 cells from HVs (n = 3), active UC (n = 2), DCC (n = 3), DCNC (n = 3), PD-1 colitis (PDC; n = 2), and PD-1 treated with no colitis (PDNC; n = 3). (*A*) t-stochastic neighbor embedding (t-SNE) projection of live CD45^+^ lymphocytes formed 8 transcriptionally distinct clusters. (*B*) Proportion of clusters formed from cells from each disease state, with cluster 4 (box) most common in DCC. (*C*) Canonical gene markers of each clusters used to define annotation with cluster 4 (box) expressing CD3, CD8, CD69, and CD103 consistent with T_RM_ cells. (*D*) High expression of immune checkpoint molecules on (cluster 4) CD8^+^ T_RM_ cells (*box*). (*E*) t-SNE projection of T cells, highlighted by patient group. (*F*) Distribution of CD8^+^ T_RM_ cells as shown by cells co-expressing *CD8*, *CD69*, and *ITGAE(CD103)* in *pink* (low expression) and *red* (high expression). (*G*) *Histogram* showing cells that express a canonical gene-set list for T cells (*dark blue*) were selected from the total data for analysis in *E, F, G, H, I,* and *K*. (*H*) Expression of activation markers *HLADR, GZMB*, *PRF1,* and *CD38* (to a lesser extent) overlap with the CD8^+^ T_RM_ cell zone. (*I*) Expression of *IFNG* overlaps with the CD8^+^ T_RM_ cell zone with *IFNG* being detected in UC, DCC, and PDC groups. (*J*) *Heatmap* based on all CD45^+^ cells showing the most differentially expressed genes in each patient group. (*K*) *Heatmap* based on T cells only showing differential expression between *ITGAE(CD103)*^+^ and *ITGAE*^*–*^ cells.

Single-cell analysis formed 8 main clusters (Figure 5*A*) that include T cells (clusters 2, 4, and 5), B cells (clusters 1, 6, and 8), plasmablasts (cluster 7), and monocytes (cluster 3). UC has a higher proportion of B cells and plasmablasts compared with ICI-colitis groups and HV (Figure 5*B*). The defining features of each cluster are shown in Figure 5*C* and Supplementary Table 7. Co-expression of ITGAE (CD103) and CD69 is strongest in cluster 4 and identifies cells with a T_RM_ cell phenotype. The DCC group has the highest proportion of cells with a T_RM_ cell phenotype (cluster 4; Figure 5*B*). These cells have a very high expression of immune checkpoint transcripts, including *CTLA4, PDCD1 (PD-1), TIGIT, HAVCR2(TIM-3)*, and *LAG3*, which are minimally detected or absent in the other clusters (Figure 5*D*).

We further investigated the T cell component of the scRNAseq dataset (Figure 5*E* and *G*). CD8^+^ T cells with a T_RM_ cell phenotype can be identified as cells co-expressing transcripts for *CD8A, CD8B, ITGAE*, and *CD69* (Figure 5*F*), and there is evidence for these cells expressing high amounts of *HLADR, GZMB*, and *PRF1* (Figure 5*H*). We have demonstrated by Nanostring and bulk RNASeq that IFNG signaling pathways are enriched in anti–CTLA-4/PD-1 colitis, but the source of *IFNG* message cannot be determined at a bulk RNASeq level. Using scRNASeq, we are able to confirm *IFNG* production in T cells that overlap with the T_RM_ cell zones (Figure 5*F* and *I*) and that *IFNG* production is detected in all UC, DCC, and anti–PD-1 colitis (PDC) groups (Figure 5*I*).

Heatmap analysis demonstrates that each group has a distinct set of up-regulated genes (Figure 5*J*). T cells from patients with DCC have significantly higher expression of *MT2A, S100A11, GNLY, MT1E, GZMB*, and *CCL20*. T cells from patients with PDC have significantly higher expression of *DUSP4, NR3C1, HLADPB1, HLADR85*, and *HSPA1A*. T cells from patients with UC have significantly higher expression of *SELL, CCR7, IFITM3, FAM118A*, and *FCMR*.

The defining characteristics of ITGAE^+^(CD103^+^) colonic T cells (compared to ITGAE^–^ T cells) are shown in Figure 5*K*. These cells have significantly higher expression of *CCL5, NKG7, CD8A, GZMA, TMIGD2, TNFRSF9, GZMB, GNLY, IFNG, CXCR6, LAG3*, and *TIGIT*, and lower expression of *CCR7, CD28*, and *CD4*.

**Figure 6:**
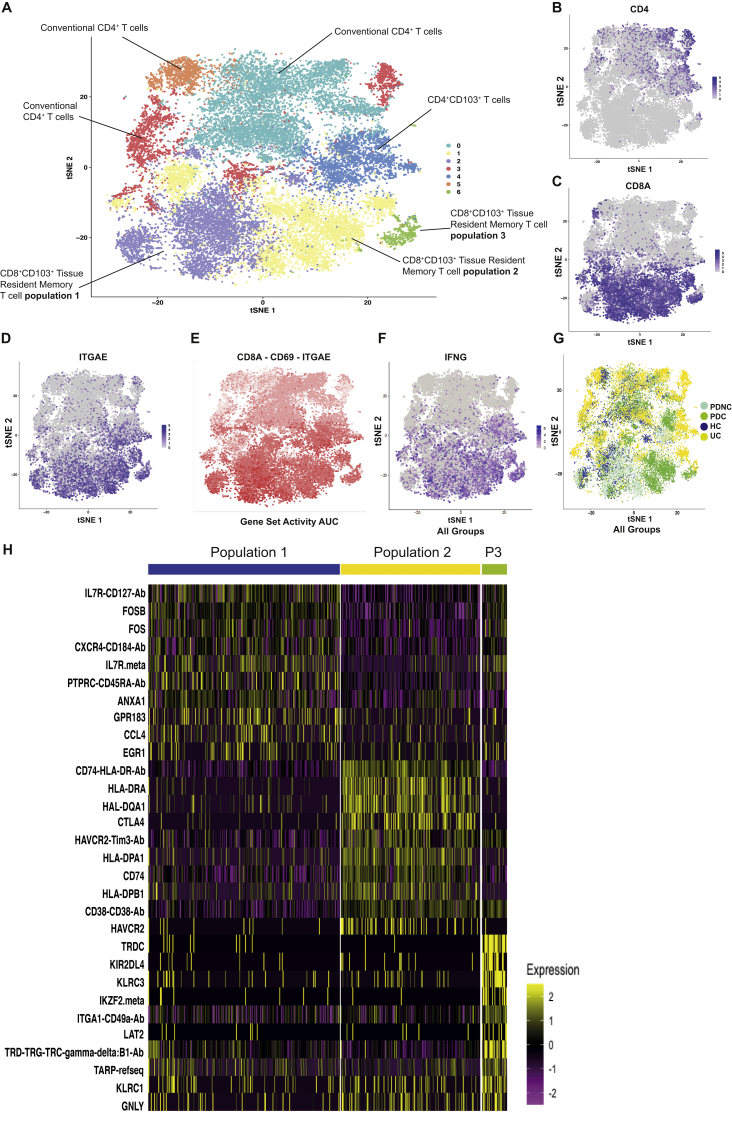
CD8^+^ T_RM_ cells express high levels of *IFNG* in PD-1–associated colitis. Data from a single cell protein and RNASeq analysis of 23,265 gut-derived T cells from HVs (n = 4), patients with active UC (n = 3), PD-1 colitis (PDC; n = 5) and PD-1 treated with no colitis (PDNC; n = 2). (*A*) t-stochastic neighbor embedding (t-SNE) projection of live T cells formed 7 distinct clusters. t-SNE plots of all groups showing expression of (*B*) *CD4*, (*C*) *CD8A*, and (*D*) *ITGAE*(CD103). (*E*) Distribution of CD8^+^ T_RM_ cells as shown by cells co-expressing *CD8*, *CD69*, and *ITGAE*(CD103) in *pink* (low expression) and *red* (high expression). (*F*) Expression of *IFNG* is shown in all groups, displaying overlap with CD8^+^ T_RM_ cell zones. (*G*) Distribution of cells based on patient groups demonstrates T cells from patients with PDC are found predominantly in the CD8^+^ T_RM_ cell zones (clusters 1 and 6). (*H*) *Heatmap* based on CD8^+^ T_RM_ cell populations 1–3 only displaying top differentially expressed genes.

To confirm our finding that CD8^+^ T_RM_ cells display high expression of *IFNG*, including in patients with PDC, we performed a second single-cell protein and RNASeq experiment, this time sorting on live CD45^+^CD3^+^ T cells (Figure 6). The cells form 7 clusters (Figure 6*A* and Supplementary Table 8), including 3 clusters of CD8^+^ T_RM_ cells (clusters 1, 2, and 6), a tissue-resident CD4^+^ T cell population (cluster 4), and 3 populations of nonresident classical T cells (clusters 0, 3, and 5). There is a clear separation of CD4^+^ (Figure 6*B*) and CD8^+^ T cell populations (Figure 6*C*), with CD103(ITGAE) predominantly overlapping with CD8^+^ T cells (Figure 6*D*). Co-expression of CD8, CD103(ITGAE), and CD69 defines the CD8^+^ T_RM_ cell clusters (Figure 6*E*). Expression of *IFNG* overlaps with CD8^+^ T_RM_ cell populations and, to a lesser degree, the CD4^+^CD103^+^ T cells (Figure 6*F*). T cells from patients with PD-1 colitis are predominantly in the CD8^+^ T_RM_ cell populations (clusters 1 and 6) and are distinct from T cells from active UC, which are predominantly in the conventional and CD4^+^ T cell zones (Figure 6*G*).

We extracted data pertaining to the 3 CD8^+^ T_RM_ cell populations (Figure 6*H*) and found that the CD8^+^ T_RM_ cell population 2, comprised mostly cells from patients with PD-1 colitis, has markedly high expression of activation markers (*HLADR, CD38*) and checkpoint molecules (*CTLA4, TIM3*). This transcriptome is distinct from CD8^+^ T_RM_ cell population 1, which has a greater representation in health and expresses high levels of *IL7R* and *CCL4*. The smaller CD8^+^ T_RM_ cell population 3 again contains unique features, including *KIR2DL4*, *KLRC3*, and includes a γδ T cell component.

### Tofacitinib Results in Rapid Resolution of Treatment-Refractory Anti–PD-1–Associated Colitis and Reversal of CD8^+^ Tissue Resident Memory T Cell Activation and Interferon-Gamma Signaling

**Figure 7:**
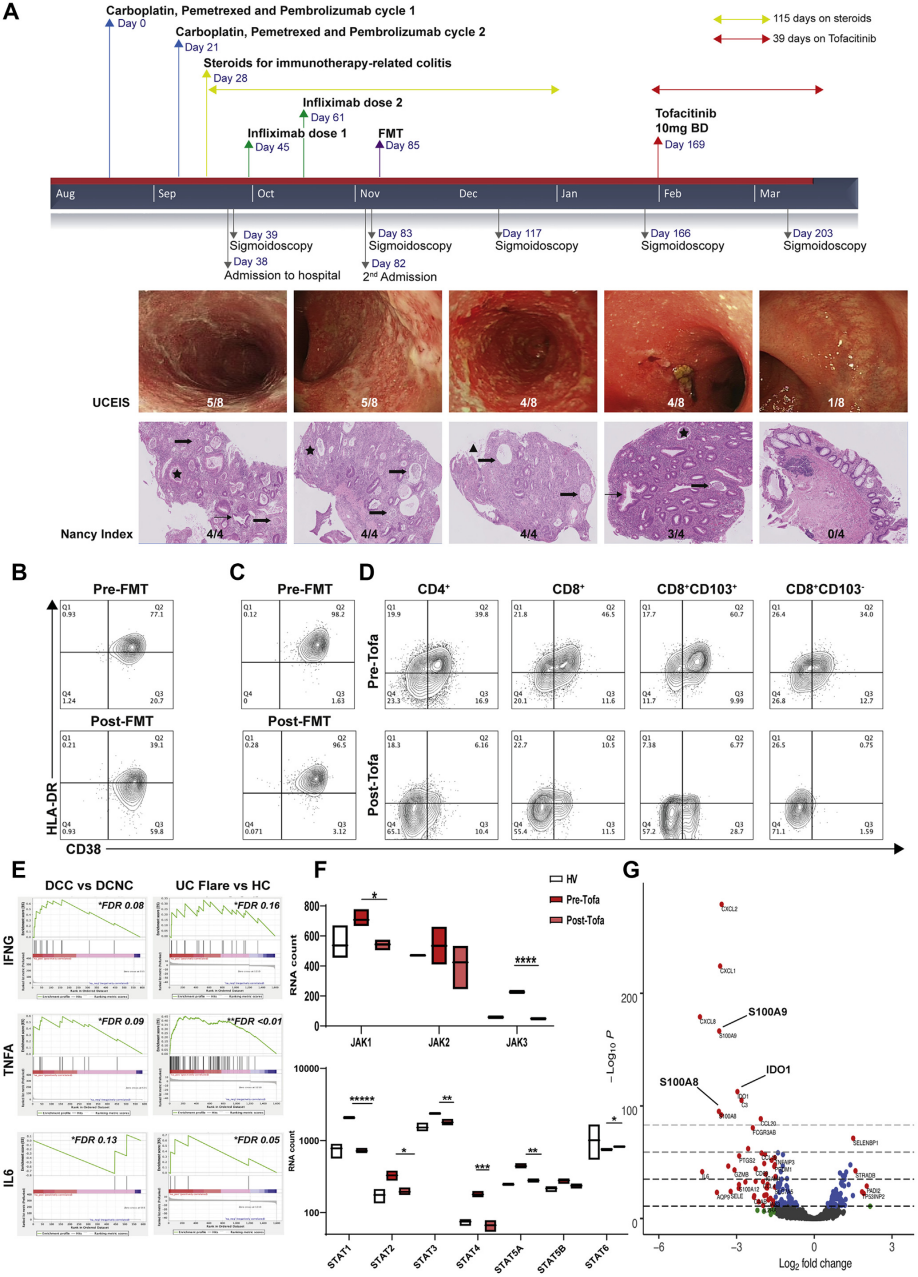
Tofacitinib results in rapid resolution of treatment-refractory ICI-colitis, and correlates with resolution of CD8^+^ T_RM_ cell activation and down-regulation of JAK/STAT signaling. (*A*) Clinical time course of a 61-year-old man with non–small-cell lung cancer treated with carboplatin, pemetrexed, and pembrolizumab. The anti–PD-1 colitis was refractory to multiple therapies. Tofacitinib resulted in prompt resolution of clinical symptoms, and endoscopic and histopathology inflammation. Tofacitinib was continued for 6 weeks. *Star*, crypt abscess; *thick arrow*, attenuated crypt; *thin arrow*, crypt architectural distortion; *triangle*, erosion. (*B*) FMT response in a previous patient with ICI-colitis, where clinical resolution was associated with normalization of CD8^+^ T_RM_ cell activation. Flow cytometry gated on live CD3^+^CD8^+^CD69^+^CD103^+^ T_RM_ cells. (*C*) Using the same donor stool, FMT did not result in resolution of clinical symptoms or resolution of CD8^+^ T_RM_ cell activation in this 61-year-old man who subsequently received tofacitinib. (*D)* Flow cytometry plots gated on live single CD45^+^CD3^+^ T cells are shown. Before tofacitinib, widespread activation of CD4^+^ and CD8^+^ T cells is evident, with the highest level of activation in CD8^+^CD103^+^ T_RM_ cell subset (61%). After 6 weeks of tofacitinib, there is resolution of T cell activation, including in the CD8^+^CD103^+^ T_RM_ cell subset (7%). (*E)* Gene set enrichment analysis of bulk RNASeq data demonstrates *IFNG* signaling pathway enrichment in ICI-colitis. (*F*) Data from Nanostring RNAplex assay from the tofacitinib-treated patient and 3 HVs. Tofacitinib results in significant down-regulation of JAK1, JAK3, STAT1, STAT2, STAT3, STAT4, and STAT5A. ∗*P <* .05; ∗∗*P* < .01; ∗∗∗*P* < .001; ∗∗∗∗*P* < .0001; and ∗∗∗∗∗*P* < .00001 by Mann-Whitney test. (*G*) Volcano plot depicting pre and post tofacitinib.

A 61-year-old man with metastatic non-small-cell lung cancer was treated with combination chemotherapy (carboplatin and pemetrexed) and pembrolizumab (see Figure 7*A*). After 2 cycles, he developed symptoms of ICI-colitis, which was confirmed endoscopically and histologically (UCEIS and Nancy score details are in Supplementary Figure 1). He did not respond to intravenous steroids or 2 doses of infliximab. We had previously seen a rapid resolution of refractory anti–CTLA-4/PD-1 colitis with FMT treatment in a different patient, with corresponding decreases in CD8^+^ T_RM_ cell activation (Figure 7*B*). We prioritized FMT over vedolizumab as he required rapid induction therapy. There was a modest initial clinical response to FMT; however, on follow-up sigmoidoscopies over the subsequent 12 weeks, he continued to have refractory colitis of an equivalent endoscopic and histologic severity. This was confirmed by flow cytometry (Figure 7*C*). Our data collectively provided us with evidence that tofacitinib, a JAK inhibitor, was a potential therapeutic option. After discussion with the patient, tofacitinib was prescribed at 10 mg twice per day, with concomitant venous thromboembolism prophylaxis.^35^ He made an immediate clinical response and achieved endoscopic and histologic remission by 5 weeks (Figure 7*A*), commensurate with resolution of activated CD8^+^ T_RM_ cells (61%–7%; Figure 7*D*). Tofacitinib was ceased after 6 weeks, and he restarted chemotherapy. He has made a good oncological response and 10 months later colitis has not recurred.

An RNAplex assay of colonic mucosal RNA pre- and post-tofacitinib therapy shows down-regulation of key JAK-STAT signaling components known to be down-stream of IFNG signaling (Figure 7*F*). The overall transcriptional response to tofacitinib therapy demonstrates down-regulation of transcripts including *S100A8, IDO1*, and *S100A9* (Figure 7*G*).

## Discussion

The profound success of ICIs has resulted in broader applications, and an increasing incidence of ICI-colitis that is frequent and has the greatest overall irAE mortality.^3^

Here we present a comprehensive analysis of ICI-colitis using multiparameter and spectral flow cytometry, RNAplex, and bulk and single-cell analysis. We find that in all patient groups, the majority of colon-derived CD8^+^ T cells are T_RM_ cells, and that in anti–CTLA-4/PD-1 colitis, the highest activation levels are seen in T_RM_ cells. CD8^+^ T_RM_ cell activation anti–CTLA-4/PD-1 colitis correlates with clinical, endoscopic, and histopathologic findings, and the response to treatment over time. In addition, CD8^+^ T_RM_ cell activation is also present in ICI gastritis, which involves a distinct epithelium and microenvironment from the colon, and this may have implications for the pathogenesis of extragastrointestinal irAEs.

We have made a direct comparison with active UC and find that the level of CD8^+^CD103^+^ T cell activation is significantly greater in the DCC group. We identify both the immunologic commonalities with UC and disparate features. In addition to differences in T cell populations, we demonstrate that B lineage populations are over-represented in the UC samples and comparatively absent in ICI groups, indicating that pathogenic B cells likely play a smaller role in ICI-colitis. The *IFNG*-response pathway is up-regulated in both UC and DCC, but with an enhanced activation in both DCC and PDC indicating that targeting this pathway may be even more effective in ICI-colitis.

Our scRNASeq experiments confirm that CD8^+^ T_RM_ cells are enriched in ICI-colitis, and display the highest proportion of immune checkpoint transcripts, including CTLA4 and PCDC1 (PD-1). This supports recently published data^21^ and provides a likely mechanism by which these cells become disproportionally and rapidly activated after ICI administration. We confirm that the production of *IFNG* clusters in the same region as CD8^+^ T_RM_ cells, extending the data from Luoma et al,^21^ which identified *IFNG* and *TNFA* up-regulation in the CD4^+^ and CD8^+^ T cells, and acting on the myeloid cellular compartment. Our analysis of ITGAE(CD103)^+^ T cells reveals an expression profile similar to CD8^+^ T_RM_ cells in vitiligo,^13^ with 5 up-regulated genes that largely relate to cytotoxicity featuring in both datasets: *GZMB, GNLY, NKG7, CCL5*, and *IFNG.* Our data suggest that in health, CD8^+^ T_RM_ cells express a homeostatic signature, including IL7R, but that in ICI-associated colitis, there is significant up-regulation of activation molecules and checkpoint molecules.

We find compelling evidence for up-regulated *IFNG* signaling in ICI-colitis, more so than *TNFA*, which is the current target of ICI-colitis rescue therapy. We present a patient with refractory ICI-colitis who we treated successfully with tofacitinib, and with robust resolution of CD8^+^ T_RM_ cell activation. This aligns with the recent case reports of successful tofacitinib therapy for treatment-refractory ICI-colitis.^22^^,^^23^ We acknowledge that a series of cases cannot conclusively determine a treatment’s large-scale efficacy or safety. IFNG signaling is a well-established pathway in tumor control. Early work in murine models found that neutralizing IFNG interferes with tumor rejection in immunocompetent hosts.^36^ Mice lacking *IFNG* signaling components IFNGR1 or STAT1 develop a higher percentage of tumors at a faster rate.^37^ We recognize concern that use of JAK inhibition in ICI-colitis may deactivate not only colonic but also intra-tumoral CD8^+^ T_RM_ cells, which are a key therapeutic target.^18^^,^^38^ In human melanoma, IL-7 signaling,^39^ T cell infiltration, and IFNG signaling signatures^40^ have a high association with tumor response to immune checkpoint inhibitors, and conversely defects in IFNG signaling, including loss-of function mutations in IFNGR1, JAK1, JAK2, and STAT1, are associated with resistance to checkpoint blockade.^41^, ^42^, ^43^, ^44^, ^45^ Although larger clinical trials are needed to establish the safety and efficacy of tofacitinib for ICI-colitis, our analysis provides a complete bench-to-bedside cycle: from a novel disease entity, hypothesis, and mechanistic study, to intervention with a rationally repurposed therapeutic. Our data suggest that tofacitinib may be a useful therapy in patients with refractory ICI-colitis as a salvage therapy.

Our study has limitations. We acknowledge our relatively small cohort of patients and, given the real-world nature of our study, these patients are not perfectly matched. In addition, the relatively low cell number in some of the experiments, notably the initial scRNASeq, mean that the UC and ICI-colitis comparison could bias toward commonalities. Comparison of a chronic disease (UC) with longstanding inflammatory response against an acute iatrogenic colitis may also confound the data. Although the patients with UC do have a longer median flare duration, the cohorts are otherwise well-matched in relation to inflammation severity, and previous and current therapy (see Supplementary Table 1). Future studies could aim to reduce the heterogenicity of enrolled ICI-colitis and comparator where possible, and this may require larger multicenter studies. In addition, although we attempted to study ICI-colitis CD8^+^ T_RM_ cells ex vivo, the high rates of apoptosis, in keeping with down-regulated Bcl-2 expression (see Supplementary Figure 2), made this challenging. We suggest cluster 4 in our scRNASeq experiment is likely numerically under-represented, given our flow cytometry results were conducted on fresh tissue.

There remain many unanswered questions, including what factors drive a subset of patients to develop ICI-colitis, leaving others unaffected. We postulate that activated CD8^+^ T_RM_ cells in ICI-colitis are responding to commensal or pathogenic microbes, and that this results in high levels of cellular activation and IFNG signaling that propagates downstream and widespread tissue activation. Furthermore, the use of FMT for treatment of ICI-colitis (for which we describe one treatment success) implies that replacement of the microbiome may remove the instigating antigen.^6^

Our work has identified that *IFNG*-producing CD8^+^ T_RM_ cells are a cellular hallmark of ICI-colitis. This has important implications for targeted therapy for ICI-colitis, as evidenced by the successful application of a JAK inhibitor. These findings also suggest that medications that specifically target CD103 may prove effective therapy, and note monoclonal antibody targeting the β7 integrin chain. Finally, our data on CD8^+^ T_RM_ cell activation in both colon and gastric epithelium may have broader relevance for other extra-gastrointestinal irAEs.

## Figures and Tables

**Supplementary Table 1:** Clinical Characteristics of DCC, DCNC, UC, PDC, PDNC, and HV Groups

Variable	DCC	DCNC	UC	PD-1 checkpoint inhibitor-related colitis	PD-1 checkpoint inhibitor no colitis	HVs
n	15	10	10	7	5	22
Age, *y*, median (IQR)	69 (59–70)	60 (54–70)	44 (34–49)	64 (61–71)	72 (70–74)	67 (50–73)
Sex, *male*, n (*%*)	10 (67)	3 (30)	6 (60)	6 (86)	4 (80)	12 (55)
Cancer			NA			NA
Melanoma stage, n						
IIIB	1	1		0	2	
IIIC	1	2		0	0	
IIID	0	0		1	1	
IV	11	7		0	0	
Renal	2	0		3	1	
Lung	0	0		3	1	
Serum LDH,^a^*U/L*, median (IQR)	259 (222–301)	289 (259–331)	NA	216 (210–235)	210 (205–227)	NA
Distribution of UC	NA	NA	4 Pancolitis 6 Left-sided colitis	NA	NA	NA
Time from UC diagnosis to endoscopy, *mo*, median (IQR)	NA	NA	50 (41–161)	NA	NA	NA
Time from diarrhoea onset to endoscopy, *d*, median (IQR)	10 (6–29)	9 (3–26)	27 (1–49)	16 (9–17)	12 (11–13)	NA
Patients on steroids at endoscopy, n (*%*)	12 (80)	5 (50)	3 (30)	2 (29)	0 (0)	NA
Time on steroids at endoscopy, *d*, median (IQR)	21 (28–63)	16 (2–27)	45 (40–54)	32 (19–45)	0 (0)	NA
Colitis treatment, n		NA			NA	NA
Mesalazine	0		7	0		
Prednisolone	15		6	7		
Intravenous methylprednisone	11		0	0		
Azathioprine	0		3	0		
Mycophenolate mofetil	1		0	0		
Infliximab	8^b^		3^c^	2^d^		
Adalimumab	0		1	0		
Vedolizumab	4^d^		3^e^	0		
FMT	1^d^		0	1^d^		
Tofacitinib	0		0	1^d^		
Colectomy	0		0	0		
Endoscopic findings (UCEIS score), n						
0	0	10	0	1	5	22
1	4	0	2	0	0	0
2	1	0	0	2	0	0
3	3	0	2	0	0	0
4	4	0	1	3	0	0
5	1	0	5	1	0	0
6	2	0	0	0	0	0
7	0	0	0	0	0	0
8	0	0	0	0	0	0
Histologic findings, n						NA
Focal active colitis	2	0	0	0	0	
Collagenous colitis	1	0	0	2	0	
Lymphocytic colitis	1	0	0	1	0	
IBD-like colitis	7	0	10 (UC)	1	0	
Infectious/NSAID-like colitis	3	0	0	3	0	
Normal	1	10	0	0	5	
Nancy score, n						NA
0	0	10	0	1	5	
1	3	0	1	1	0	
2	5	0	3	3	0	
3	5	0	4	2	0	
4	2	0	2	0	0	

IQR, interquartile range; LDH, lactate dehydrogenase; NA, not applicable; NSAID, nonsteroidal anti-inflammatory drug.

gene Nanostring experiment analyzing RNA extracted from the gastrointestinal tract of HVs (n = 8), patients with active UC (n = 5), anti–CTLA-4/PD-1 colitis (DCC; n = 9) and anti–CTLA-4/PD-1 treated with no colitis (DCNC; n = 8) are shown. *Plots* show differentially expressed genes in (*i*) UC vs HV and (*ii*) DCC vs DCNC and DCC vs UC.

a Normal range, 90–235 U/L.

b 2 of 8 patients were on infliximab at the time of endoscopy and 6 had it subsequently.

c 2 of 3 patients were on infliximab at the time of endoscopy and 1 had had it prior.

d Biologic (or FMT) was given after the endoscopy in patients.

e 2 of 3 patients were on vedolizumab at the time of endoscopy and 1 had had it prior.

**Supplementary Table 2:** Patient Samples and Experiment Allocations

Sample	Category	Flow cytometry		Fluorescence microscopy	Bulk RNASeq	Nanostring	scRNASeq	Single-cell protein-RNASeq
		Basic panel	Extended panel					
HV3664	HV	•						
HV3536	HV	•						
HV3665	HV	•			•			
HV3684	HV	•			•			
HV3523	HV	•			•	•		
HV3947	HV	•				•		
HV3379	HV	•				•		
HV3132	HV	•			•	•		
HV3530	HV				•			
HV3085	HV				•	•		
HV3667	HV				•			
HV3384	HV				•	•		
HV3127	HV				•	•		
HV3452	HV				•			
HV3687	HV				•			
HV3859	HV				•		•	
HV3947	HV				•		•	
HV4166	HV				•		•	•
HV3367	HV					•		
HV4407	HV							•
HV2885	HV							•
HV4116	HV							•
IBDU3087	UC	•		•	•	•		
UC1-2865	UC	•		•	•	•		
UC7	UC	•			•	•		
UC8	UC	•		•	•	•		
UC9	UC	•				•		
UC2-3105	UC				•			
UC4-3814	UC	•		•	•	•	•	•
UC6-3817	UC	•		•	•	•	•	
UC1975	UC							•
IBD240	UC							•
DCC4051	CTLA-4/PD-1 colitis	•	•	•	•	•		
PRISE3	CTLA-4/PD-1 colitis	•	•					
DCC4442	CTLA-4/PD-1 colitis	•	•					
PRISE14	CTLA-4/PD-1 colitis	•	•					
DCC3188	CTLA-4/PD-1 colitis	•		•	•	•		
DCC3237	CTLA-4/PD-1 colitis	•		•	•	•		
DCC3700	CTLA-4/PD-1 colitis	•		•	•	•		
DCC4158	CTLA-4/PD-1 colitis	•	•			•	•	
PRISE7	CTLA-4/PD-1 colitis	•	•		•	•	•	
DCC4054	CTLA-4/PD-1 colitis	•			•	•	•	
DCC4055	CTLA-4/PD-1 colitis	•				•		
DCC4059	CTLA-4/PD-1 colitis	•			•	•		
LP11	CTLA-4/PD-1 colitis				•			
LP13	CTLA-4/PD-1 colitis				•			
LP16	CTLA-4/PD-1 colitis				•			
2PRISE3	CTLA-4/PD-1 TX no colitis	•	•		•	•	•	
DCNC4149	CTLA-4/PD-1 TX no colitis	•	•	•		•	•	
DCNC3662	CTLA-4/PD-1 TX no colitis	•		•	•	•	•	
DCNC4158	CTLA-4/PD-1 TX no colitis			•				
DCNC4159	CTLA-4/PD-1 TX no colitis	•		•		•		
DCNC3723	CTLA-4/PD-1 TX no colitis	•		•	•	•		
PRISE1	CTLA-4/PD-1 TX no colitis	•	•		•	•		
PRISE11	CTLA-4/PD-1 TX no colitis	•	•					
PRISE12	CTLA-4/PD-1 TX no colitis	•	•		•	•		
DCNC-3237	CTLA-4/PD-1 TX no colitis				•	•		
CC2-3126	PD-1 colitis						•	•
CC8-3660	PD-1 colitis						•	
GI4378	PD-1 colitis							•
GI4303	PD-1 colitis							•
PRISE5	PD-1 colitis							•
GI4445	PD-1 colitis							•
3592	PD-1 TX no colitis						•	
PRISE13	PD-1 TX no colitis						•	
PRISE2	PD-1 TX no colitis						•	
GI4069	PD-1 TX no colitis							•
PRISE15	PD-1 TX no colitis							•

**Supplementary Table 3:** Clinical Characteristics of DCG and HV Groups

Characteristic	DCG	HV
n	4	7
Age, *y*, median (IQR)	69 (65–73)	66 (51–72)
Sex, *male*, n (*%*)	3 (75)	3 (43)
Cancer, n		NA
Melanoma stage IV	3	
Renal	1	
Serum LDH,^a^*U/L*, median (IQR)	256 (230–309)	NA
Time from symptom onset to endoscopy, *d*, median (IQR)	18 (14–33)	NA
Patients on steroids at endoscopy, n (*%*)	2 (50)	NA
Time on steroids at endoscopy, *d*, median (IQR)	11 (10–12)	NA
Gastritis treatment, n		NA
Prednisolone	4	
Infliximab	1^b^	

DCG, dual checkpoint inhibitor-related gastritis; IQR, interquartile range; LDH, lactate dehydrogenase; NA, not applicable.

a Normal range, 90–235 U/L.

b Biologic was given after the endoscopy in patient.

## Supplementary Methods

### Illustrative Figures

Illustrative figures were created with the assistance of BioRender.com

### Flow Cytometry

Flow cytometry on freshly isolated gut mononuclear cells was performed using a near infra-red live/dead stain (Invitrogen) and the following monoclonal antibodies: CD3 PE-CF594(UCHT1), CD4-BV650(SK3), CD8-AF700(SK1), CD38-FITC(HIT2) (all, BD Biosciences), CD45RO-BV510(UCHL1), CD69-BV785(FN50), CD103-BV605(Ber-ACT8), and HLA-DR-BV711(L243) (Biolegend). Isotype control stains were performed using mouse IgG2aκ and IgG1κ (BD Biosciences). This work was performed on a 3-laser LSR Fortessa X-20 (BD Biosciences).

An extended T_RM_ cell phenotyping panel was run using the aforementioned near infra-red live/dead stain and following antibodies: CD4-BV650(OKT4), CD38-AF488(HIT2), CD45RO-BV510(UCHL1), CD69-BV785(FN50), CD103-BV605(Ber-ACT8), Bcl-2-AF647(100), CTLA-4-PE(BN13), CXCR6-APC(KO41E5), HLA-DR-BV570(L243), Ki-67-PerCP-Cy5.5(Ki-67), LAG3-BV421(11C3C65), PD-1-AF647(EH12.2H7) (Biolegend), CD8-AF532(RPA-T8), TIGIT-PERCP-EF710(46-9500-42) (ThermoFisher Scientific), CD3-PECF594(UCHT1), granzyme B-BV421(GB11), and TIM-3-BB515(7D3) (BD Biosciences). Granzyme B, Ki-67, and Bcl-2 staining was performed using an intracellular and intranuclear permeabilization kit (ThermoFisher). The extended panel was performed on an Aurora spectral analyzer (Cytek, Freemont, CA).

Data were analyzed using FACSDIVA software, version 8.0.1 (BD Biosciences). Compensation was calculated before each experiment. Approximately 150,000 lymphocytes (50,000 live T cells) were analyzed per colon biopsy. Gating strategies are shown in Figure 1*Aix–x*. Lymphocyte populations are reported as a proportion of parent populations. Specifically, live CD3^+^ T cells are reported as percentage of total live lymphocytes (as defined by side scatter vs forward scatter, CD45^+^, and exclusion of viability dye). CD4^+^ and CD8^+^ T cells are reported as a percentage of live T cells. CD8^+^CD103^+^CD69^+^ T_RM_ cells are reported as a percentage of total CD8^+^ T cells. Activated (HLA-DR^+^CD38^+^) cells are reported as percentage of memory CD4^+^ or CD8^+^ T cell populations.

### Nanostring

Custom 10-gene spike in set: CD69, ITGAE(CD103), RUNX3, NR4A1, MKI67, LAMP1, TNFSF9(CD137), CXCL16, HAVCR2(TIM-3), and IL2RA.

### Single-Cell Protein and RNA Sequencing Expression

Viably cryopreserved gut-derived mononuclear cells were thawed in a 37°C water bath before washing and quantitation. The number of cells were normalized across all subjects. Individual patient samples were labeled with oligo-conjugated sample tag antibodies (Sample Multiplexing Kit; BD Biosciences) by incubating for 15 minutes at room temperature followed by the addition of anti-human CD45-APC (Clone HI3-; BD Biosciences) and CD3-PE-CF594 (Clone UCHT1; BD Biosciences) for 15 minutes at room temperature. Cells were washed twice with 15 mL phosphate-buffered saline and centrifuged at 500*g* before combining into a single tube and staining with SyberGreen viability dye (Sigma).

### Single-Cell Protein and RNA Sequencing Complementary DNA Library Preparation and Sequencing

cDNA was amplified for 10 cycles using the predesigned T cell expression panel (BD Biosciences) in combination with supplementary custom primer panel. In total, the primer set used in this assay contained 565 probes targeting 534 different genes. The resulting PCR1 products were purified using AMPure XP magnetic beads (Beckman Coulter) and the respective messenger RNA (mRNA) and AbSeq/Sample tag products were separated based on size-selection, using different bead ratios (0.7X and 1.2X, respectively). The purified mRNA and Sample tag PCR1 products were further amplified (10 cycles), and the resulting PCR2 products purified by size selection (0.8X and 1.2X for the mRNA and sample tag libraries, respectively). The concentration, size, and integrity of the resulting polymerase chain reaction products was assessed using both Qubit (High Sensitivity dsDNA kit; Thermo Fisher) and the Agilent 4200 Tapestation system (High Sensitivity D1000 screentape; Agilent). The final products were normalized to 2.5 ng/μL (mRNA), 1 ng/μL (sample tag), and 0.2 ng/μL (AbSeq) and underwent a final round of amplification (6 cycles for mRNA and sample tag and 7 cycles for AbSeq) using indexes for Illumina sequencing to prepare the final libraries. Final libraries were quantified using Qubit and Agilent Tapestation and pooled (approximately 23:73:4% mRNA/AbSeq/Sample tag ratio) to achieve a final concentration of 5 nM. Final pooled libraries were spiked with 15% PhiX control DNA to increase sequence complexity and sequenced (150-bp paired-end) on a NovaSeq sequencer (Illumina).

### Single-Cell RNA and Protein: Data Analysis and Quality Control

After filtering the PhiX reads, we achieved a final coverage of 567 × 10^6^ reads. The FASTQ files obtained from sequencing were analyzed following the BD Biosciences Rhapsody pipeline (BD Biosciences), as described previously.^1^ The distribution-based error correction–adjusted molecule counts were imported and the expression matrices analyzed using R package.

We applied *scater* package to filter out single-cell profiles that were outliers for any metric, as low-quality libraries.^2^ Technical noise was modeled using the *scran* package^3^ based on the optimal number of principal components.^4^

Sub-setting was performed to select cells expressing *CD8A*, *CD8B*, and *CD4*, *ITGAE*, and *IFNG.* A principle component analysis was run with 7 canonical clusters selected for downstream analysis using the Seurat package.^5^ An integrated analysis of all merged data was performed using defined canonical clusters. Plots were generated using ggpubr (version 0.2) and customizing ggplot2.^6^

We applied area under the curve and bimodal distribution to separate the distributions and evaluate the strength of enrichment of each reference cell with genes in an indicated cell.
